# Emergency Spatiotemporal Shift: The Response of Protein Kinase D to Stress Signals in the Cardiovascular System

**DOI:** 10.3389/fphar.2017.00009

**Published:** 2017-01-24

**Authors:** Brent M. Wood, Julie Bossuyt

**Affiliations:** Department of Pharmacology, University of California, Davis, DavisCA, USA

**Keywords:** protein kinase D, GPCR, cardiovascular disease, heart failure, metabolism, oxidative stress

## Abstract

Protein Kinase D isoforms (PKD 1-3) are key mediators of neurohormonal, oxidative, and metabolic stress signals. PKDs impact a wide variety of signaling pathways and cellular functions including actin dynamics, vesicle trafficking, cell motility, survival, contractility, energy substrate utilization, and gene transcription. PKD activity is also increasingly linked to cancer, immune regulation, pain modulation, memory, angiogenesis, and cardiovascular disease. This increasing complexity and diversity of PKD function, highlights the importance of tight spatiotemporal control of the kinase via protein–protein interactions, post-translational modifications or targeting via scaffolding proteins. In this review, we focus on the spatiotemporal regulation and effects of PKD signaling in response to neurohormonal, oxidant and metabolic signals that have implications for myocardial disease. Precise targeting of these mechanisms will be crucial in the design of PKD-based therapeutic strategies.

## Introduction

**FIGURE 1:**
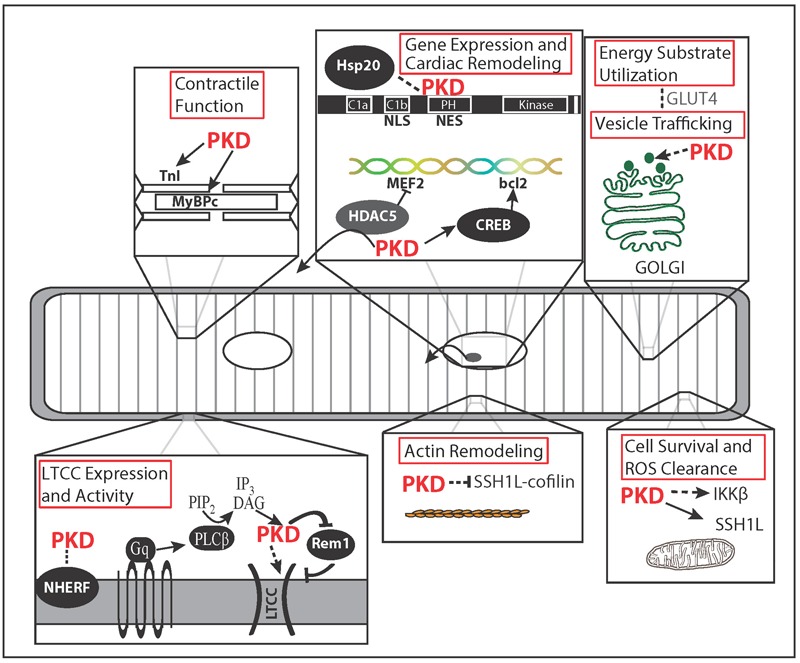
**Microdomain Functions of Protein Kinase D (PKD).** Spatial regulation of PKD activity allows for specificity of PKD function. PKD localizes to the membrane after Gα_q_ signaling initiates DAG production via phospholipase C. There PKD phosphorylation of Rem1 results in greater membrane insertion and activity of L-type Ca^2+^ channels (LTCC). PKD targeting to the myofilaments and its substrates, TnI and MyBPC, results in decreased myofilament Ca^2+^ sensitivity, increased cross bridge cycling rate, and increased contraction tension. Nuclear localization and exclusion sequences within PKD regulate nuclear localization potentially along with the chaperone protein Hsp20. PKD regulates growth through phosphorylation of HDAC5 and CREB. In non-cardiovascular cell types, PKD is linked to Golgi organization, membrane-vesicle fusion, and secretion. In the heart, vesicle trafficking is potentially linked to increased glucose uptake during pacing through GLUT4 membrane translocation. Of the actin remodeling effects described for PKD, only the Slingshot 1L (SSH1L)-cofilin signaling axis has been demonstrated in myocytes and linked to cell survival. PKD-IKKβ signaling has also been linked to cell survival and reactive oxygen species (ROS) clearance. Dashed arrows indicate pathways or functions not shown in cardiovascular cell types.

Protein kinase D (PKD) is emerging as a key signaling hub in the heart affecting excitation-contraction coupling, gene expression, cell survival, and metabolism (**Figure 1**). There is compelling evidence from numerous *in vitro* and *in vivo* studies for a prominent PKD role in activating gene programs driving adverse morphological and functional changes in cardiomyopathy, prompting considerable excitement in its therapeutic potential. *In vivo*, cardiac-specific expression of constitutively active PKD1 caused pronounced dilated cardiomyopathy; and PKD expression and activity is increased in failing mouse, rat, rabbit and human myocardium vs. non-failing (Harrison et al., 2006; Bossuyt et al., 2008; Taglieri et al., 2014). Genetic studies including genome wide association studies also linked mutations in the *PRKD1* gene to syndromic congenital heart defects and body mass index (an established risk factor for cardiovascular disease; Speliotes et al., 2010; Comuzzie et al., 2012; Graff et al., 2013; Shaheen et al., 2015; Sifrim et al., 2016). In loss-of-function studies, cardiac-specific PKD1 knockout mice (cKO) proved remarkably resistant to cardiac hypertrophy and fibrosis in response to pressure overload or chronic administration of both isoproterenol and angiotensin (Fielitz et al., 2008). Interestingly, beneficial effects of PKD were also found recently. PKD activation enhanced tolerance to ischemia/reperfusion injury (Xiang et al., 2011, 2013b). PKD activation also mitigated lipid accumulation, insulin resistance and maladaptive remodeling in diabetic cardiomyopathy (Dirkx et al., 2014) although other groups found PKD inhibition enhanced cardiac function in that setting (Liu et al., 2015; Venardos et al., 2015). These observations underscore the need for better insight into the mechanistic basis of PKD actions in the heart and their role in cardiovascular disease.

## PKD Structure and Activation Mechanisms

**FIGURE 2:**
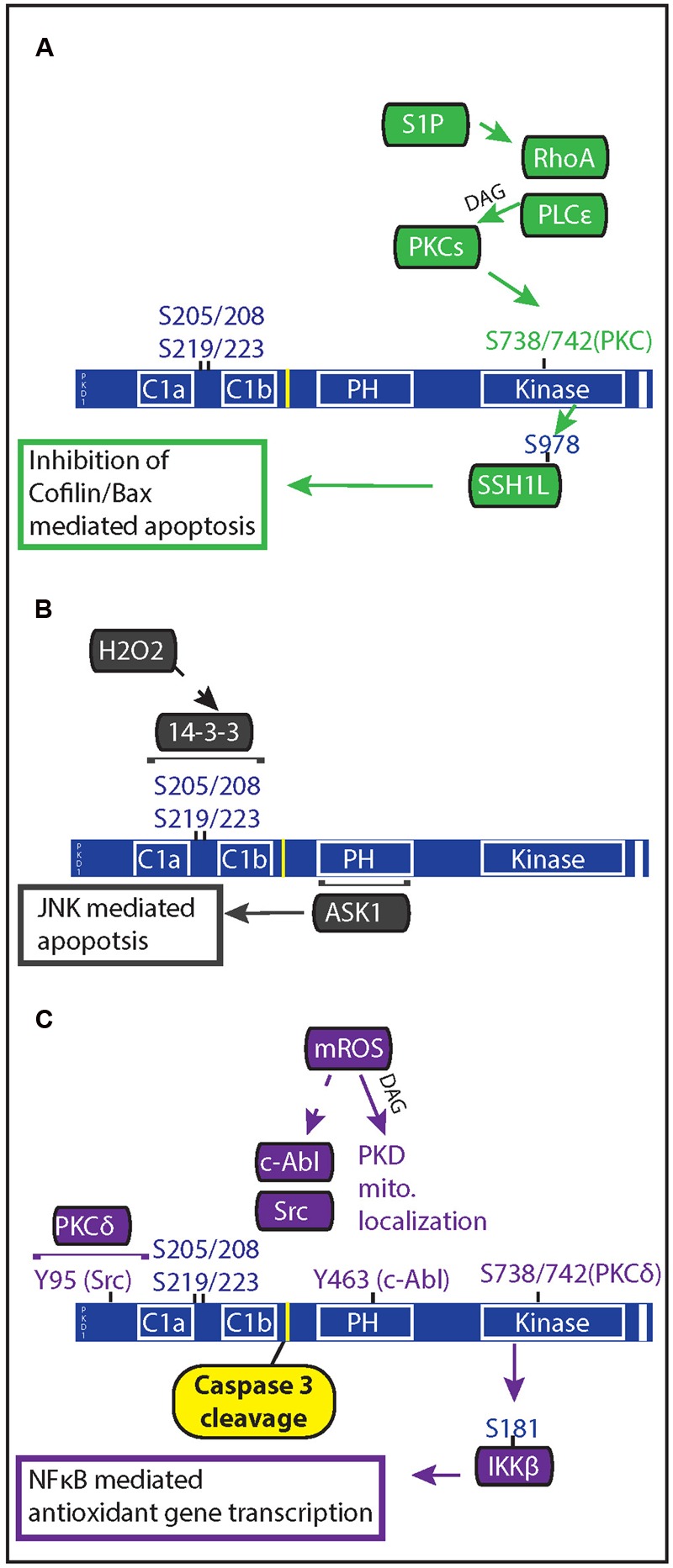
**Oxidative stress regulation of PKD mediated anti-apoptotic signaling.** PKD both regulates and is regulated by apoptotic signals. **(A)** In response to ischemia reperfusion injury, cardiac myocytes release of sphingosine 1 phosphate which acts through Gα_12/13_-coupled receptors to activate RhoA. Rho A mediates PKC and PKD activation through activation of phospholipase C𝜀. PKD phosphorylation of the phosphatase slingshot 1 L (SSH1L) inhibits the ability of SSH1L to activate cofilin, preventing it from translocating with Bax to the mitochondria. **(B)** Generation of mitochondrial ROS results in DAG production through phospholipase D1 and PKD localization at the mitochondria. Once at the mitochondria, c-Abl and Src phosphorylation of PKD allows for PKCδ activation of PKD. Active PKD goes on to phosphorylate IκBα resulting in NFκB gene transcription of MnSOD, which allows for greater mitochondrial clearance of ROS. **(C)** Increased oxidative stress through H_2_O_2_ treatment causes PKD translocation to the nucleus and results in 14-3-3 interaction with two phosphorylated serine pairs in the linker region between the C1 domains of PKD. Apoptosis signal-regulating kinase 1 (ASK1) associates with the PH domain of PKD1, leading to ASK1 activation of c-Jun N-terminal kinase (JNK); and JNK-mediated apoptosis in endothelial cells.

There are three highly homologous PKD isoforms: PKD1/PKCμ, PKD2, and PKD3/PKCν (Valverde et al., 1994; Hayashi et al., 1999; Sturany et al., 2001). Of these, PKD1 is the most studied in cardiomyocytes. While the PKD isoforms have a similar modular structure, they do exhibit some variability which may account for some of the distinct functions of PKD isoforms that are emerging (Ellwanger and Hausser, 2013). PKDs consist of a highly conserved C-terminal catalytic domain (consisting of motifs required for ATP/substrate-binding and catalysis) and an N-terminal regulatory domain (**Figure 2**). The structural and enzymatic properties of the catalytic domains are quite distinct from those of the protein kinase A, G, and C (AGC) serine/threonine kinase subfamily (Hanks, 2003). Thus PKD isoforms have been reassigned to the Ca^2+^/calmodulin-dependent protein kinase (CaMK) superfamily.

The autoinhibitory N-terminal domain contains twin cysteine-rich zinc-finger motifs (C1a and C1b) and a pleckstrin homology (PH) domain. The tandem C1 motifs function as lipid-binding membrane-targeting modules without a calcium requirement (similar to their counterparts in PKCs). The differential lipid-binding preferences of the individual motifs result in their different roles in intracellular targeting of PKD, although non-lipid-binding interactions contribute too (e.g., with the nuclear localization signal in C1b; Rey et al., 2001a; Oancea et al., 2003; Wang et al., 2003; Chen et al., 2008). The C1 motifs also modulate kinase activity, however, the extent of catalytic inhibition depends upon the isoform. In PKD1, deletion of C1a, C1b or both results in constitutive activity; whereas in PKD2, little to no effect on PKD activity is seen with C1 deletion (Iglesias and Rozengurt, 1999; Auer et al., 2005).

The PH domain likewise suppresses catalytic activity, an effect which is relieved by tyrosine phosphorylation within the PH domain (Storz et al., 2003), protein interactions with the PH domain (e.g., with PKCη or G_βγ_ (Jamora et al., 1999; Waldron et al., 1999) and/or by activation loop phosphorylation (Waldron and Rozengurt, 2003). The PH domain also contains a nuclear export signal for PKD in the Crm1-dependent nuclear export pathway (Rey et al., 2001a; Auer et al., 2005) but unlike other PH domains, it displays only low affinity toward phospholipids (Wang et al., 2003). It is still unclear whether the PH domain contributes to the membrane-docking interactions of PKD.

Finally, the C-terminal autophosphorylation sites in PKD1 and 2 are part of a post-synaptic density-95/discs large/zonula occludens-1 (PDZ) binding motif, which is absent in PKD3 (Sanchez-Ruiloba et al., 2006). This raises the possibility of isoform-specific and phosphorylation-dependent interactions with PDZ proteins (Rybin et al., 2009) and might constitute a more common mechanism to activate a select subset of kinase targets. For instance the PDZ scaffold protein NHERF1 [linked to the trafficking of ion transporters, receptor tyrosine kinases and G protein coupled receptors (GPCRs)], was shown to interact specifically with PKD1 and 2 (Kunkel et al., 2009). Interestingly, NHERF1 association was capable of influencing the spatiotemporal dynamics of PKD signaling. In NHERF1-expressing HeLa cells, histamine-induced PKD1 activation at NHERF1 scaffolds is faster, more sustained, and smaller in magnitude than cytosolic PKD1 activation. The functional relevance of the PDZ-motif dependent regulation of PKD signaling has yet to be considered in cardiovascular tissues.

The structural modules of PKDs are intricately linked to the complex regulation of PKD activity and diversity of PKD actions. A wide array of stimuli can activate PKD including GPCR agonists, growth factors and cytokines (Rozengurt et al., 2005; Steinberg, 2012). In the classical activation mechanism, first described by the Rozengurt group, PKD is relieved from its basal autoinhibited state as follows: receptor stimulation promotes DAG accumulation, directly activating and colocalizing PKD at lipid membranes with allosterically activated PKCs. PKCs then *trans*-phosphorylate PKD at two highly conserved serine residues in the activation loop of the catalytic domain (S744 and S748 in mouse PKD1), relieving autoinhibition by the PH domain. PKD then autophosphorylates a cluster of sites in the C1a-C1b interdomain region and at S916 at the extreme C-terminus (mouse PKD1 nomenclature; not present in PKD3), resulting in altered binding partners and localization within the cell. The calcium-independent, novel PKCs (nPKCs: δ, 𝜀, η, and 𝜃) are the dominant PKCs involved in this process (Rey et al., 2001b, 2004; Brandlin et al., 2002; Yuan et al., 2002; Waldron and Rozengurt, 2003). However, in endothelial cells, calcium-dependent PKCα plays a role in vascular endothelial growth factor (VEGF)-induced PKD activation (Wong and Jin, 2005). Of note, PKCδ and 𝜀 have been linked to cardiomyocyte hypertrophy and death (Dorn and Force, 2005). PKC activity may also facilitate PKD “release” from the membrane (Rey et al., 2001b). The membrane translocation of PKD and subsequent redistribution to different subcellular compartments is considered a hallmark of PKD activation and has been used as a surrogate marker of enzyme activation in a cellular context (similar to PKCs).

This simple model of PKD activation is insufficient to explain the spatiotemporal dynamics of PKD activity in all cell types or even in response to all GPCRs. More recent studies indicate additional auto- and *trans-*phosphorylation reactions (by PKC and other kinases) and other regulatory mechanisms (e.g., proteolytic cleavage and docking interactions) can influence PKD signaling efficiency and specificity (reviewed in Steinberg, 2012). The functional impact of phosphorylation patterns also appears more complex. For instance, under prolonged Gαq activity each residue of the PKD activation loop is regulated by different mechanisms, namely transphosphorylation for S744 and autophosphorylation for S748 (Jacamo et al., 2008; Sinnett-Smith et al., 2009; Waldron et al., 2012). Likewise S916 phosphorylation has often been used as a marker of PKD activation, however, under some conditions it may be phosphorylated after PKD inactivation, or remain unphosphorylated while the kinase is active (Storz et al., 2004; Sanchez-Ruiloba et al., 2006; Rybin et al., 2009; Qiu et al., 2014). Additionally, both S916 and the activation loop phosphorylation were found after treatment with PKD inhibitors such as CID and Gö 6976. These phosphorylation events are uncoupled from PKD activation, as both inhibitors effectively prevent PKD catalytic activity (Kunkel and Newton, 2015). Thus, the activation loop or S916 phosphorylation state is not always equivalent to kinase activity and should not be used as the sole readout of PKD activation.

Interestingly, while the regulatory role of phosphorylations has been extensively studied, to date the effect of other post-translational modifications on PKD function has not been assessed. Moreover only a fraction of PKD-activating signaling pathways, PKD substrates and functions have been validated in cardiomyocytes (**Table 1**). Putative PKD substrates generally conform to the (L/V/I)X(R/K)XX(S/T) target motif which favors an aliphatic amino acid at the -5 position and a basic amino acid at the -3 position (Nishikawa et al., 1997; Franz-Wachtel et al., 2012). While some key cardiac PKD targets have been identified (e.g., class IIa histone deacetylases (HDACs) and troponin I) (**Figure 1**), it remains unknown to what extent the related CaMKII and PKD share effector targets {such as ion channels, Ca^2+^ handling proteins [phospholamban (PLB), ryanodine receptor (RyR)]}. Elucidation of the molecular mechanisms of PKD regulation, its effectors and their functional relevance in the healthy and diseased heart are major challenges to be met.

**Table 1:** Protein Kinase D target phosphorylation sites and verified functions in cardiovascular cell types.

Target	Phosho-site	Function	Agonist/Model	Cell/tissue type	Species	Source
Rem1	S18	Increased LTCC trafficking to the membrane and activity	Phenylephrine	Neonatal and adult ventricular myocytes	Rat	Jhun et al., 2012
eNOS	S1179	Increased NO synthesis	VEGF, PDBu	Aortic endothelial cells	Cattle	Aicart-Ramos et al., 2014
HDAC5	S259, S498	Nuclear export and MEF2 upregulation	PE, ET-1, Fetal Bovine Serum	Neonatal and adult ventricular myocytes	Rabbit	Vega et al., 2004; Bossuyt et al., 2008
CREB	S133	Increased Bcl2 expression	Thrombin and *P. multocida toxin* (rat), Gαq overexpression (mouse)	Neonatal ventricular myocytes and fibroblasts (rat), adult whole ventricle (mouse)	Rat, Mouse	Ozgen et al., 2008
Telethonin	S157, S161	Maintenance of T-tubule organization and calcium transient dynamics	Constitutively phosphorylated endogenously/*In vitro* kinase assay, Adenovirus expression	Adult ventricular myocytes, whole heart	Rat	Candasamy et al., 2014
Tn I	S22, S23	Reduction of myofilament calcium sensitivity	ET-1	Intact and skinned adult ventricular myocytes	Rat	Cuello et al., 2007
MyBPc	S302	Acceleration of cross-bridge kinetics	±PKD	Skinned ventricular myocytes	Mouse	Bardswell et al., 2010
MyBPc	S315	Increase in maximal calcium-activated contraction tension	WT/cMyBPc KO myocytes ±PKD	Permeabilized adult ventricular myocytes	Rat	Dirkx et al., 2012a
SSH1L	S978	Protection from oxidative stress response of Cofilin 2	Sphingosine 1-phosphate	Cardiomyocytes and whole left ventricles	Mouse	Xiang et al., 2013b

## Neurohormonal Stress-Dependent PKD Signaling

Neurohumoral adaptations in response to cardiac stress, initially compensating, ultimately fuel maladaptive cardiac remodeling and deteriorating cardiac performance (Bisping et al., 2014; Spaich et al., 2015; Tham et al., 2015). PKD signaling has been linked to virtually all basic cellular processes involved in remodeling, such as hypertrophy, cell death, fibrosis, angiogenesis, and inflammation. The neurohumoral storm mediators (mainly endothelin, angiotensin, and catecholamines) are all known triggers of spatial and temporal shifts in PKD activity. In cardiomyocytes, G_q_-coupled GPCRs activate PKD isoforms via phospholipase Cβ production of DAG and PKC but stimulus-specific differences exist. For example, PKD1 activity following α1-adrenergic receptor (α1-AR) stimulation is entirely PKC-dependent, whereas only the initial phase of endothelin (ET)-triggered PKD activity requires PKC (Haworth et al., 2000; Vega et al., 2004; Guo et al., 2011). The spatiotemporal dynamics of PKD activation also differ in adult cardiomyocytes: although phenylephrine (PE, an α1-AR agonist) and ET trigger comparable global PKD activation, PE induces transient sarcolemmal PKD recruitment and activation followed by nuclear import and ET prompts persistent sarcolemmal translocation and activity (Bossuyt et al., 2011). The PKD isoforms are also activated in an agonist-specific manner: norepinephrine (NE, an α/β-AR agonist) selectively activates PKD1 in neonatal myocytes and cardiac fibroblasts, whereas ET, thrombin and platelet derived growth factor (PDGF) favor PKD2/3 activation (Guo et al., 2011; Qiu and Steinberg, 2016). The molecular mechanisms underlying the differences between these two seemingly similar Gq-coupled receptors (GqR) remain to be identified.

Initial reports regarding β-AR modulation of PKD signaling were conflicting. The Olson group saw no effect of β-AR or PKA stimulation of PKD phosphorylation or activity (Harrison et al., 2006). In contrast, A kinase anchoring protein (AKAP)-Lbc was found to function as a scaffold for PKA and PKC, facilitating PKD1 activation and the transduction of hypertrophic responses (Carnegie et al., 2004, 2008). Conversely, others examining global PKD1 activity reported suppression of PKD activity by β-AR agonists or PKA (Haworth et al., 2011; Sucharov et al., 2011). Some of these conflicting reports likely reflect that global PKD1 measurements do not necessarily capture discrete pools of PKD1 signaling (Qiu and Steinberg, 2016). Indeed, specific examination of PKD microdomain signaling revealed β-AR signaling triggers both local nuclear signaling and inhibits GqR-mediated PKD1 activation by preventing its intracellular translocation (Nichols et al., 2014). Fine-tuning PKD responsiveness to GqR-agonists occurred via PKA-dependent phosphorylation of PKD S427. In this regard PKD S427 serves as an integration point of β-AR and GqR stimuli, which could be particularly relevant in heart failure progression where the β-AR pathway is desensitized.

The profibrotic mineralocorticoid aldosterone and angiotensin also activate PKD1 signaling in cardiomyocytes, suggesting a role for PKD in the control of cardiac fibrosis (Tsybouleva et al., 2004; Iwata et al., 2005). PKD1 cKO mice were dramatically resistant to fibrosis (Fielitz et al., 2008). Growing evidence from the cancer field also implicate PKD in cell proliferation and extracellular matrix remodeling via control of matrix metalloproteinase (MMP) expression and activity (Ha et al., 2008; Eiseler et al., 2009; Biswas et al., 2010; Yoo et al., 2011; Durand et al., 2016). PKD also promotes aldosterone production in adrenal cells, introducing the possibility that PKD contributes to a positive feedback loop that promotes fibrosis (Romero et al., 2006; Chang et al., 2007; Shapiro et al., 2010; Olala et al., 2014).

### Neurohormonal Regulation of Plasma Membrane Signaling

At the sarcolemma, enigma homolog 1 scaffolds PKD1 to increase Ca^2+^ current of voltage-gated Ca^2+^ channels in response to α1-AR but not β-AR stimulation (Maturana et al., 2008). PKD phosphorylation of Serine 1884 in the L-type Ca^2+^ channel (LTCC) results in increased open probability of the channel (Aita et al., 2011). PKD1 also regulates LTCC trafficking and function indirectly via phosphorylation of the GTP-binding protein Rem1 (for Rad and Gem-related) at S18. This relieves Rem1 inhibition of LTCCs, resulting in greater T-tubule expression and a corresponding increase in Ca^2+^ current density (Jhun et al., 2012).

Protein Kinase D1 has also been implicated in the phosphorylation and regulation of constitutive nitric oxide synthases (endothelial and neuronal NOS; Aicart-Ramos et al., 2014; Sanchez-Ruiloba et al., 2014). In endothelial cells, VEGF activation of PKD promotes vasodilation and angiogenesis via S1179 phosphorylation, and activation, of eNOS. Numerous signaling cascades and kinases target this regulatory site to boost NO production, including Akt. The importance of PKD as a regulatory kinase of eNOS and vascular tone was confirmed *in vivo*, where inhibition of PKD dramatically reduced VEGF-induced dilation of the carotid artery. Given its role in control of cell proliferation, migration and angiogenic gene expression (Liu et al., 2014; Ren, 2016), PKD signaling is seen as a key driver of angiogenesis with interesting therapeutic possibilities. Several studies have already shown pan-KD inhibition is beneficial in cancer models [where both the tumor cells and angiogenesis are targeted (Lavalle et al., 2010; Borges et al., 2015; Tandon et al., 2015)]. However, given the plethora of signaling cues that modulate angiogenesis in physiological and pathological conditions, the precise role of PKD isoforms in these processes should be worked out.

### Neurohormonal Regulation of Nuclear Signaling

The role of PKD in transcriptional regulation is well-documented. PKD is involved in the regulation of several transcription factors such as the activating enhancer binding protein-2 (AP2; Kondo et al., 2011), T-cell factor (TCF; via beta catenin; Jaggi et al., 2008), Snail1 (Eiseler et al., 2012), cAMP-response element binding protein (CREB; Johannessen et al., 2007; Ozgen et al., 2008), myocyte enhancer factor2 (MEF2; Vega et al., 2004) and NFκB (nuclear factor κ-light-chain-enhancer of activated B-cells; Holden et al., 2008). However, only CREB and MEF2 regulation have been confirmed in the heart. Here activation of PKD by Gq leads to CREB serine 133 phosphorylation and the induction of CRE-responsive genes such as Bcl-2 contributing to cell survival (Ozgen et al., 2008). Low levels of oxidative stress also promote CREB phosphorylation, but this is associated with decreased CREB abundance and no change CREB target gene transcription (Ozgen et al., 2009). The importance of PKD-dependent CREB phosphorylation in cardiac remodeling processes is still unclear.

Protein Kinase D also influences gene expression by modulating the epigenetic machinery, specifically the transcriptional repressors class II HDACs. The Olson and McKinsey labs firmly established PKD isoforms as HDAC kinases (McKinsey and Olson, 2005). Following activation and translocation to the nucleus, PKD associates with and phosphorylates the 14-3-3 binding sites on the HDAC protein. This unmasks a nuclear export sequence on HDAC, culminating in crm1-dependent nuclear export of the HDAC-chaperone protein complex (McKinsey et al., 2001). The release of class II HDACs from MEF2 allows for histone acetyltransferases (HATs) to associate with MEF2 inducing chromatin relaxation and transcriptional activation of fetal cardiac genes. PKD regulation of HDACs exemplifies an emerging theme in PKD regulation of its substrates: PKD target phosphorylation alters association with 14-3-3 chaperone proteins and consequently intracellular location of its substrates.

In contrast to CaMKII, each PKD isoforms can phosphorylate all of the class IIa HDACs (HDAC4, 5, 7, and 9) but their functional redundancy is not fully addressed and may be signal-dependent (Ellwanger and Hausser, 2013). In B lymphocytes, disruption of both PKD1 and 3 was required to block HDAC7 phosphorylation in antigen receptor signaling (Matthews et al., 2006). In the PKD1 cKO mice, fetal gene activation and stress-induced hypertrophy is blunted, suggesting that neither PKD2 and PKD3, nor CaMKII can fully compensate for the loss of PKD1 (Fielitz et al., 2008).

A number of regulatory interactions for PKD-HDAC signaling axis have also been identified. Disruption of the small heat shock protein 20 (hsp20)-PKD interaction, which chaperones nuclear translocation of PKD1, inhibited nuclear import of PKD and β-AR induced hypertrophy (Sin et al., 2015). In microvascular endothelial cells, PKD1/HDAC7 signaling to FoxO1 initiates proangiogenic and proarteriogenic transcription by repression of CD36 transcription (Ren et al., 2016). Four-and-a-half LIM domain proteins (FHL) 1 and 2, while not actual PKD targets, were identified as novel PKD binding partners that differentially facilitate neurohormonal activation of PKD (FHL1 for ET, and FHL2 for both ET and α1-AR agonists). Curiously, PKD regulation by FHL proteins affected HDAC5 phosphorylation but not MEF2 activation (Stathopoulou et al., 2014). Graeme Carnegie demonstrated that the AKAP-Lbc (AKAP13) scaffold protein facilitates hypertrophic PKD-HDAC signaling (Carnegie et al., 2008). This scaffolding protein, located at the nuclear envelope and cytoskeleton, nucleates PKCα, PKCη, PKA, and PKD, and promotes Rho activation (Carnegie et al., 2004). Gene-trap mice expressing an AKAP-Lbc variant that abolishes the PKD interaction (AKAP-Lbc-ΔPKD) exhibited blunted cardiac hypertrophy in response to Ang/PE treatment or pressure overload (Taglieri et al., 2014; Johnson et al., 2015), in agreement with the PKD1cKO mice. Unlike the PKD1cKO mice, the AKAP-Lbc-ΔPKD mice had greater collagen deposition and displayed an accelerated progression to cardiac dysfunction. This study further highlights the compartmentalization aspect of PKD signaling and suggests a critical *in vivo* role for AKAP-Lbc-anchored PKD signaling in compensatory hypertrophy development. In contrast, the Smrcka group proposes that the perinuclear mAKAP-PLC𝜀 complex, which generates DAG from PI4P in the Golgi apparatus in close proximity to the nuclear envelope, is the crucial scaffold complex to regulate activation of nuclear PKD and hypertrophic signaling pathways (Zhang et al., 2013). The discordant result might be explained by signal-specific pathways but both groups included ET as stimulus in their study.

### Neurohormonal Regulation of Sarcomeric Signaling

Besides the long term nuclear effects, PKD signaling may also contribute to excitation-contraction coupling regulation via effects on sarcomeric proteins. A number of PKD substrates have been identified thus far: the titin-cap protein telethonin (Candasamy et al., 2014), troponin I (TnI; Cuello et al., 2007) and Myosin Binding Protein c (MyBPc; Bardswell et al., 2010; Dirkx et al., 2012a). Upon phosphorylation of TnI at Serine 22 and 23, the sites also targeted by PKA, myofilament Ca^2+^ sensitivity is reduced. Phosphorylation of MyBPc at Serine 302 and Serine 315 accelerates crossbridge cycling kinetics and increases maximal Ca^2+^-activated contraction tension.

Force generation was not modulated by the coordinated phosphorylation of TnI and MyBPc. Instead, active PKD can affect force generation by increasing Ca^2+^ current via increased surface expression of LTCCs. Paradoxically, α1-AR stimulation with PE, elicits a triphasic response in both the left ventricle (LV) and right ventricle (RV), but an overall positive inotropic response in LV trabeculae and an overall negative inotropic response in RV trabeculae (Wang et al., 2006). The overall response of each ventricle does not correspond to the response of individual myocytes, however, as α1-AR signaling causes both a positive and negative inotropic effect in individual myocytes of both ventricles that is independent of which α1-AR subtype is stimulated (Chu et al., 2013). In what is likely a compensatory mechanism, heart failure shifts the overall negative response to PE in the RV to a positive one (Wang et al., 2010). Although PKD is a key target of α1-AR signaling it is unknown whether α1-adrenergic-PKD signaling is involved in either the heterogeneous inotropic response of individual myocytes or the switch of inotropy during HF.

Despite a well-established nuclear translocation effect from the membrane to the nucleus, the spatiotemporal dynamics of PKD at its sarcomeric targets has not been assessed. It is unclear whether the active PKD that phosphorylates TnI and MyBPc originates from the same pool that is destined for the nucleus, or whether there is a separate scaffolded pool of PKD available to respond locally within the sarcomeres.

## Oxidative Stress-Dependent PKD Signaling

Evanescent reactive oxygen species (ROS) are continuously produced by metabolic and cellular processes and may accumulate with ischemic injury or upon various pathological insults, determining whether ROS act as modulators of signal transduction pathways and physiological processes or as mediators of cellular injury. Extensive data have implicated ROS in the development and progression of cardiovascular disease, diabetes, cancer and neurodegenerative disorders. Not so surprisingly, PKD is also activated by oxidative stress. Downstream of PKD activation, three pathways were shown to impact cell survival: via NFκB, RhoA, and c-Jun N-terminal kinase (JNK) (**Figure 2**) (Storz and Toker, 2003a; Waldron et al., 2004; Zhang et al., 2005; Xiang et al., 2011). To promote these oxidative stress responses, PKD acts in the nucleus and mitochondria.

### Oxidative Stress Regulation of Nuclear signaling

In endothelial cells, PKD1 was found to be a critical mediator of H_2_O_2_- but not TNF-induced apoptosis signal-regulating kinase 1 (ASK1) activation of JNK (Waldron et al., 2004; Zhang et al., 2005). Here H_2_O_2_ triggered PKD activation and translocation from the membrane to the perinuclear region, where it associated with ASK1, before progressing to the nucleus. The ASK1-PKD1 interaction (via the PKD PH domain) was critically dependent on 14-3-3 binding to PKD at two pairs of phosphorylated serines (205/208 and 219/223). The H_2_O_2_ –induced ASK-JNK activation and endothelial cell apoptosis was blocked with PKD inhibition or siRNA knockdown. Whether ASK1 is also modulated by PKD in cardiomyocytes or whether PKD1 regulation of ASK1 activity is via direct phosphorylation (as shown for CaMKII) is still unknown.

### Oxidative Stress Regulation of Mitochondria to Nucleus Signaling

The Storz group identified a pathway where PKD activation by mitochondrial ROS led to induction of antioxidative genes through activation of NFκB. In HeLa cells, mitochondrial oxidative stress promoted local DAG production by phospholipase D1 evoking PKD translocation to the mitochondria (Cowell et al., 2009). PKD activation at the mitochondria was dependent upon the tyrosine kinases c-Abl and Src (Storz et al., 2003). c-Abl phosphorylation of Tyrosine 463 within the PKD PH domain led to loss of autoinhibition and Src phosphorylation of PKD Tyrosine 95. These phosphotyrosines create a docking site for PKCδ via its C2 domain and subsequently phosphorylation of Serine 744/748 in the activation loop. PKCδ recognizes this phosphorylated tyrosine and associates with PKD resulting in phosphorylation at Serines 744 and 748 (Storz et al., 2004). Active PKD then phosphorylates inhibitor of NFκB kinase β (IKKβ) at Serine 181, which allows for IκBα degradation and NFκB activation (Storz and Toker, 2003b). In the nucleus NFκB promotes SOD2 gene transcription which encodes the mitochondrial protein manganese depend superoxide dismutase (MnSOD; Storz et al., 2005). The subsequent detoxification of ROS resulted in increased cell survival but perturbation of any components in this pathway led to increased cell death. It is not clear whether this pathway operates in cardiac myocytes. It should also be noted that growth factor-dependent PKD1 signaling cascades do not activate NFκB nor induce MnSOD, underscoring the impact of contextual cues and specific post-translational modifications of PKD1 on achieving unique cellular outcomes.

Protein Kinase D1-mediated protection from oxidative stress may also be mediated via RhoA signaling (Xiang et al., 2013a). During ischemia-reperfusion injury cardiomyocytes release the cardioprotectant sphingosine 1-phosphate (S1P; Vessey et al., 2009). The signaling cascade initiated by S1P via its Gα_12/13_-coupled receptor, produce guanine nucleotide exchange factors for RhoA activation which in turn activates PLC𝜀 (Wing et al., 2003; Xiang et al., 2013b). The surge in DAG, activates PKD1 which then phosphorylates and inhibits Slingshot 1L (SSH1L), a phosphatase/activator of cofilin (actin depolymerization factor). S1P thus attenuates cofilin translocation to mitochondria and association with the pro-apoptotic BAX (bcl-2-associated X protein), preserving mitochondrial integrity and cell survival in the face of oxidative stress (Klamt et al., 2009). In mice with genetic deletion of PKD1 or PLC𝜀, S1P-mediated cardioprotection against ischemia/reperfusion injury was reduced (Xiang et al., 2013b). The PKD-slinghot-cofilin signaling axis is one of many PKD-mediated pathways regulating actin dynamics described thus far (Olayioye et al., 2013). Determining if PKD also fulfills this role in cardiac cell types may shed light on cytoskeletal and actin rearrangement seen with heart failure.

## Metabolic Stress-Dependent PKD Signaling

Investigation of PKD signaling in pancreatic and muscle tissue is a developing field that can potentially reveal novel molecules and pathways that regulate metabolism and diabetes development. PKD signaling has already been implicated in the regulation of energy substrate utilization and insulin secretion. Intriguingly the PRKD1 gene locus has also been associated with increased body mass index (Speliotes et al., 2010; Comuzzie et al., 2012; Graff et al., 2013; Shaheen et al., 2015; Sifrim et al., 2016). Graff et al. (2013) further found a stronger impact during adolescence than in older adults which could reflect PKD expression decline in adulthood (as shown in rodent hearts; Haworth et al., 2000; Vega et al., 2004; Guo et al., 2011). Although obesity is the best predictor for type 2 diabetes development the few studies examining PKD function in diabetic models provided conflicting results as both PKD overexpression and inhibition were linked to improved cardiac function.

### Metabolic Stress Regulation of Golgi to Membrane Signaling

In pancreatic β-cells, PKD1 is essential for glucose-stimulated insulin secretion signaling downstream of the long-chain fatty acid (LCFA) receptor GPR40 (Ferdaoussi et al., 2012). In these cells PKD activity is negatively controlled by MAPKp38δ phosphorylation (at Serines 397/401). Accordingly, p38δ KO mice displayed high constitutive PKD activity and were protected against high-fat-feeding induced insulin resistance and oxidative stress–induced βcell failure (Sumara et al., 2009).

In the heart, PKD is crucial for contraction-induced translocation of the glucose transporter type 4 (GLUT4), an essential step to stimulate cardiac glucose uptake during increased energy demand (Luiken et al., 2008). The underlying mechanism is not a direct effect of ATP/AMP levels, as with AMP-activated kinase (AMPK), but rather a result of contraction-induced ROS production, association of death-activated protein kinase (DAPK) and subsequent PKD activation (Dirkx et al., 2012b). AMPK represents the other obligatory signaling branch regulating GLUT4 translocation in cardiomyocytes, but unlike PKD, AMPK also mediates increased CD36 membrane translocation, allowing for LCFA to enter the cell (Dirkx et al., 2012b). Indeed, PKD1 cKO myocytes, despite having intact AMPK levels, do not increase their glucose uptake with pacing and their LCFA uptake is enhanced (Dirkx et al., 2014). Conversely, mice expressing constitutively active PKD1 (caPKD1) have 1.5 times the level of basal glucose uptake and a more pronounced increase in uptake in response to pacing than WT myocytes (Dirkx et al., 2014). Distinct roles for PKD and AMPK are also seen in rat cardiomyocytes with low glucose uptake induced by high insulin or high palmitate culture conditions. Either PKD or AMPK overexpression prevents loss of insulin-stimulated glucose uptake. But while overexpression of PKD in cardiomyocytes prevents lipid loading, AMPK overexpression promotes retention of insulin-stimulated Akt signaling (Steinbusch et al., 2013). Given its independence from the insulin signaling axis, the PKD signaling pathway represents a strategic option to increase glucose uptake in the insulin-resistant diabetic heart.

Conversely, PKD also regulates cardiomyocyte lipoprotein lipase (LPL) secretion, which hydrolyzes lipoproteins at the vascular lumen (Kim et al., 2008). In diabetes, the observed increase in LPL facilitates the switch to the disproportionate use of fatty acids (FA) which ultimately leads to excessive cardiac lipid accumulation and dysfunction (Wang and Rodrigues, 2015). The action of PKD1 to regulate LPL-mediated triglyceride accumulation was discovered in mice treated with diazoxide to decrease insulin secretion and cause hyperglycemia. The mechanism relied on heat shock protein 25 dissociation from PKCδ, permitting PKCδ association with and activation of PKD. Active PKD then promoted vesicular trafficking and release of LPL. A subsequent study recapitulated these findings in rats using low dose streptozotocin (STZ) to induce moderate hypoinsulinemia (Kim et al., 2009). Whereas in rats with severe hypoinsulinemia induced by a higher dose of STZ, LPL activity at the vascular lumen was diminished. The loss of LPL stimulation was attributed to caspase-dependent cleavage of PKD (resulting in a modest increase in basal activity of the kinase, but severe limitation of its maximal activation). These observations contrast with the finding that cardiac lipid overload and insulin resistance are largely prevented in caPKD mice fed a high fed diet (Dirkx et al., 2014). Clearly further investigation is needed to clarify the beneficial and pathogenic roles of PKD in regulating energy substrate utilization.

### Metabolic Stress Regulation of Nuclear Signaling

Several groups have performed proof-of-concept studies targeting PKD in diabetic models. Some groups have targeted PKD-mediated cardiac remodeling in diabetic cardiomyopathy, while others have attempted to capitalize on PKD modulation of fuel selection. Using the early stage type 2 diabetes model *db/db* mice, which have a point mutation in one of the leptin receptor genes, administration of the pan-PKD inhibitor CID755673 for 2 weeks effectively inhibited PKD isoforms, suppressed the gene expression signature of PKD activation and enhanced diastolic and systolic function as well as reduced heart weight. The improvement in cardiac indices was independent of effects on glucose homeostasis, insulin action and body composition (Venardos et al., 2015). Liu et al. likewise found that the observed cardiac fibrosis, apoptosis, diastolic dysfunction and ER stress were all related to PKD activation in a rat diabetic model achieved by a combined low dose STZ and high fat diet (4 weeks). Irbertesan treatment ameliorated cardiac remodeling and function in this model, which was attributed to inhibition of PKD activation and ER stress function (Liu et al., 2015).

A third model, mice on a chronic (8 weeks) high fat diet, displayed the characteristic insulin resistance, cardiac switch to LCFA and lipid deposition, and LV concentric hypertrophy but also reduced PKD expression and activity levels (Dirkx et al., 2014). Interestingly, applying the high fat diet to caPKD1 mice rescued their baseline dilated cardiomyopathy phenotype (without inducing hypertrophy) and also prevented cardiac lipid overload and insulin resistance. The only two mutual responses to high fat diet of WT and caPKD mice were reduced protein levels of PKD and decreased levels of HDAC5 phosphorylation. Of note, despite the decreased PKD expression, PKD activity levels were unchanged, suggesting that not just the expression but also the location and/or substrate of PKD is altered by metabolic stress. Some of these conflicting reports likely reflect variations in the animal models and our poor understanding of the temporal and complex role of PKD signaling in metabolism. There is a tendency to oversimplify the mechanistic basis of PKD effects to the context of single downstream targets (e.g., cardiac remodeling to the HDAC5-Mef2 axis), which is clearly not the only mode of action. Much remains to be learned about the precise regulation and effectors of PKD signaling in response to metabolic stress.

## Concluding Remarks

The preceding studies convincingly implicate PKD in the pathogenesis of multiple cardiovascular risk factors and the subsequent cardiovascular disease, but are just the tip of the iceberg. We are still far from understanding the intricacies of PKD signaling especially in the context of complex disease pathways. Many questions remain unanswered regarding the specific beneficial and pathogenic roles of PKD and the role of specific PKD isoforms therein. Tissue- and isoform-specific KO mice, as well as the development of isoform-specific PKD inhibitors should provide important breakthroughs. Another critical challenge is the vexing state of affairs regarding straightforward analysis of PKD activation state. Proteomic approaches identifying the relevant regulatory post-translational modifications and interactions would provide key insight into PKD signaling specificity and regulation. This approach could also guide the identification and verification of PKD effector targets in cardiovascular cells. Finally, PKD represents an enticing therapeutic target for a number of pathologies including cancer. Given the already apparent complexities of the functions and interactions of PKD isoforms, it is unlikely that a “one size fits all” PKD inhibitor will be a viable therapeutic strategy. Any successful strategy will have to minimize undesirable off-target effects but also adverse on-target effects in other tissues.

## Author Contributions

BW wrote the initial draft of the manuscript and JB edited for content and form.

## Conflict of Interest Statement

The authors declare that the research was conducted in the absence of any commercial or financial relationships that could be construed as a potential conflict of interest.
